# Promoter DNA methylation analysis reveals a novel diagnostic CpG-based biomarker and RAB25 hypermethylation in clear cell renel cell carcinoma

**DOI:** 10.1038/s41598-017-14314-y

**Published:** 2017-10-27

**Authors:** Yinmin Gu, Yi Ming Zou, Danqing Lei, Yuanjie Huang, Weidong Li, Zengnan Mo, Yanling Hu

**Affiliations:** 1 Life Sciences Institute, Guangxi Medical University, Nanning, Guangxi 530021 China; 2 Department of Mathematical Sciences, University of Wisconsin-Milwaukee, Milwaukee, WI 53201 USA; 3 Center for Genomic and Personalized Medicine, Guangxi Medical University, Nanning, Guangxi 530021 China; 4 Guangxi Colleges and Universities Key Laboratory of Biological Molecular Medicine Research, Guangxi Medical University, Nanning, Guangxi 530021 China

## Abstract

Clear-cell renal cell carcinoma (ccRCC) is a common aggressive urinary malignant tumor that cannot be easily diagnosed at an early stage. The DNA methylation occurs within promoter before precancerous lesion plays a pivotal role that could help us in diagnosing and understanding ccRCC. In this study, based on a whole-genome promoter DNA methylation profiling, we used shrunken centroids classifier method to identify a CpG-based biomarker that is capable of differentiating between ccRCC tumor and adjacent tissues. The biomarker was validated in 19 ccRCCs and three public datasets. We found that both CYP4B1 and RAB25 are downregulated with promoter hypermethylation and CA9 is upregulated with promoter hypomethylation, and we validated their mRNA differential expressions in 19 ccRCCs and 10 GEO datasets. We further confirmed that hypermethylated RAB25 is inversely correlated with its mRNA level. Log-rank test showed that ccRCC patients with low levels of CA9 promoter methylation had a higher survival rate. This reveals clinically a potential biomarker for use in early detection for ccRCC, and provides a better understanding of carcinogenesis.

## Introduction

Renal cell carcinoma (RCC) has the highest mortality rate among urinary malignant tumors, and it represents 2–3% of human malignant neoplasms^1,2^. Clear-cell renal cell carcinoma (ccRCC), which accounts for 80–90% of RCC, is the most frequent and aggressive subtype^2^. One third of RCC cases are asymptomatic at early stages and are already metastatic when diagnosed, which leads to a 95% mortality rate^3^. Conventional diagnostic methods such as imaging studies and ultrasound or computed tomography-guided biopsies have a limited reliability in distinguishing RCC in early stages^4^, thus there is a strong need to identify ccRCC biomarkers for early tumorigenesis. There are many known risk factors for RCC, such as smoking, obesity, hypertension and so on, but it can be argued that genetic variant is a major factor^5^. The initiation and progression of carcinoma are connected with changes in the DNA coding sequences, and they are also hereditable variants in phenotypes or expressions of genes due to epigenetic events^6^. Evidences have suggested that DNA methylation plays a key role in ccRCC as one of the most common epigenetic changes^7,8^.

DNA methylation patterns are prone to CpG islands, most of which are found in the proximal promoter regions of almost 60% of human genes in the mammalian genomes^9^. DNA methylation alters biological functions through regulating the stabilization of genomic sequences or the expressions of genes^10^. It can restrain transcription factor bindings, alter chromatin structures, and prevent transcription factors to access gene promoters^11^. Gene promoters can be accessible to regulatory units that are important cis-acting regulatory elements, which initiate and regulate genes’ expressions^11^. Despite the fact that CpG islands methylations also exist within gene bodies and deserts, their relation to gene expressions is not yet clear^9^. Gene promoters can be accessible to regulatory units that are important cis-acting regulatory elements, which initiate and regulate genes’ expressions. The fact that DNA methylation frequently occurs as preneoplastic changes motivated researchers to study methylation as a potential detection indicator for early-stage or potentially premalignant diseases^12^. From a practical point of view, the use of DNA methylation as a possible detection indicator also has other advantages. For examples, DNA is a more stable molecule than RNA or protein chemically, DNA methylation is tissue-specific, and CpGs have a high degree of sensitivity^13^.

In this study, we profiled promoter-region DNA methylation in 265 ccRCC primary tumors and 133 adjacent tissues with Illumina HumanMethylation450 from The Cancer Genome Atlas data (TCGA), and found that a CpG-based biomarker (cg11201447, cg25247520, cg13309012, cg08995609) can efficiently distinguish ccRCCs from adjacent tissues and that RAB25 is hypermethylated in ccRCC tissues. We further validated our findings using 19 ccRCC tissues and GEO datasets. Our results provide new information about aberrant DNA methylation within the promoters of ccRCC and suggest a potential diagnostic biomarker for the disease.

## Results

### Identification of differential methylation between ccRCC and adjacent tissues

**Figure 1:**
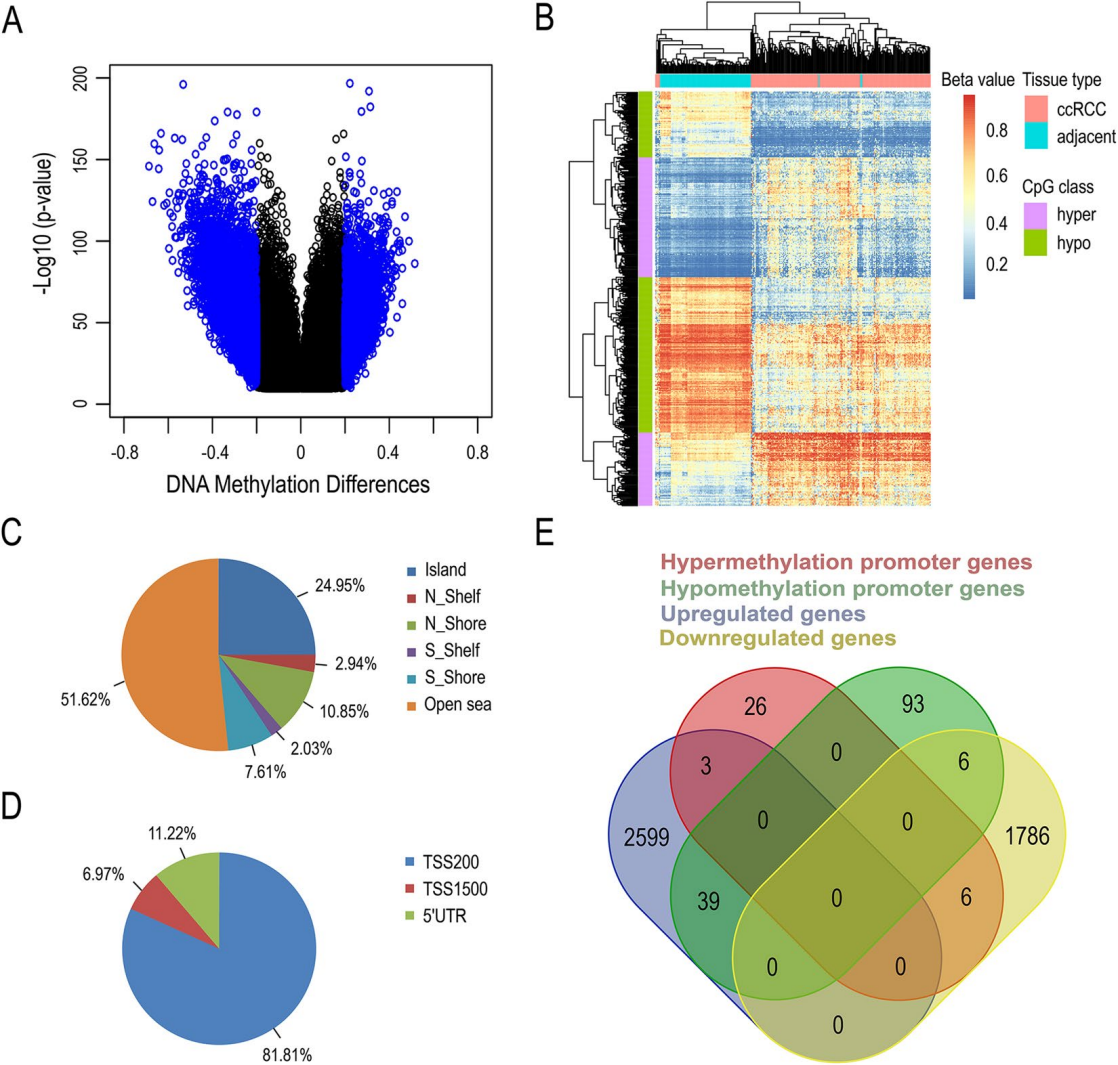
Differential methylation between ccRCC and adjacent tissue. (**A**) Volcano plot showing the distribution of CpG sites from the site-level test assessed by methylation differences and adjusted *P* values. All of CpGs are with *FDR* < 1E-10, CpGs in blue are differentially methylated more than 0.2 or less than −0.2; (**B**) Heatmap of differentially methylated CpGs within promoters between ccRCCs and adjacent tissues. Hierarchical clustering by 265 ccRCCs (pink color bar) and 133 adjacent tissues (blue-green color bar) with 986 significant CpGs within promoters (*FDR* < 1E-10, |Delta Beta| > 0.2); Red/blue gradient represents beta values of the methylation sites; (**C**) Promoter region distribution of promoter differential methylated CpG sites; (**D**) CpG island distribution of promoter differential methylated CpG sites; (**E**) Venn diagram showing the overlaps of the genes with the differential promoter methylation and mRNA regulation.

To explore the ccRCC whole-genome promoter DNA methylomes, we analyzed 267 primary ccRCCs and 133 adjacent tissues using the TCGA Illumina HumanMethylation450 microarray. After quality control and filtering, DNA methylation analysis on 265 ccRCCs, 133 adjacent tissues and 448625 probes was performed. These samples were collected between the year 1998 and 2013. The detailed clinical data was presented in Table 1. By using the linear models for microarray data (limma) approach to identify the statistically different DNA methylation status between ccRCC and adjacent tissues, 13617 CpGs were found to be significantly different (*FDR* < 1E-10, |Delta Beta| > 0.2). Compared to adjacent tissue, 4552 CpGs were hypermethylated and 9065 CpGs were hypomethylated in ccRCC (Fig. 1A). Among these, 986 CpGs were located in promoter regions (TSS200, TSS1500 and 5’UTR) (Supplementary Table 1). Unsupervised clustering analysis was used to assess the underlying methylation differences between these groups of samples. From Fig. 1B, two parted clusters can be readily observed–one contains almost entirely of adjacent samples and the other almost entirely of the ccRCC samples, indicating that the pattern of promoter DNA methylation is quite different between ccRCCs and adjacent tissues. The promoter CpG sites were primarily distributed in Open Sea (51.62%) for CpG islands and TSS200 (81.81%) for promoter regions (Fig. 1C,D).

**Table 1:** Characteristics of ccRCC samples from the TCGA data portal.

Variables	Characteristics	No. of tumors	No. of adjacents
Sex	Male	173	90
	Female	92	43
Race	White	231	128
	Black	33	4
	Asian	1	1
Age	<60	129	58
	≥60	136	75
Tumor size	T1	121	43
	T2	37	21
	T3	99	62
	T4	8	7
Lymph node	N0	113	56
	N1	8	4
	NX	144	73
Metastasis status	M0	197	104
	M1	46	29
	MX	20	0
	NA	2	0
Fuhrman grade	G1	6	0
	G2	106	43
	G3	106	59
	G4	43	30
	GX	2	1
	NA	2	0

Abbreviations: NA, Not Available; No., Number.

With the cutoff values *FDR* < 0.05 and |Delta Beta| > 0.2, 35 differentially promoter hypermethylated genes and 138 promoter hypomethylated genes were identified. Apart from protein coding genes, there are 22 pseudogenes and 23 non-coding RNA genes (Supplementary Table 2). Our finding of the most hypomethylated interleukin 8 gene in ccRCC was consistent with a published study^14^. To integrate both promoter DNA methylation and gene expression to select genes of potential biological importance for validation, RNA sequencing data from TCGA was used for analysis. The analysis results showed that 2640 genes were upregulated and 1798 genes were downregulated in 525 ccRCCs comparing to 69 adjacent tissues (*FDR* < 0.05, |log2 FC| > 1) (Supplementary Table 3). We found that the DNA methylation levels of 45 genes are inversely correlated with their mRNA levels, 39 of which are promoter hypomethylated genes that have overexpressions in ccRCC tissue. The overlaps of differentially methylated and mRNA regulated genes were shown by a venn diagram in Fig. 1E.

### Diagnostic methylation markers

**Figure 2:**
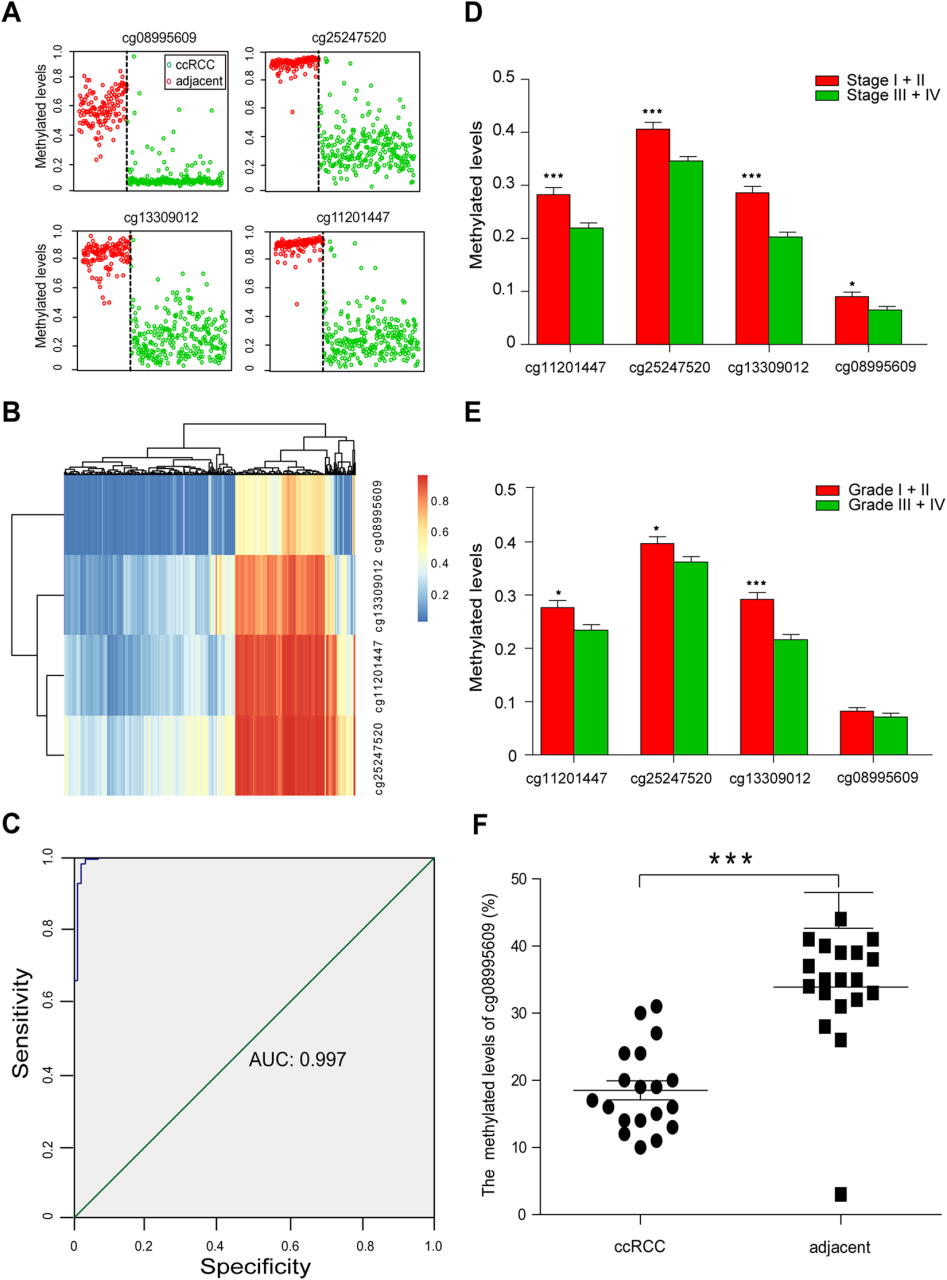
The methylated levels of cg11201447, cg25247520, cg13309012, cg08995609. (**A**) Methylated levels of the 4 CpGs in 265 ccRCCs (green circle) and 133 adjacent normal tissues (red circle) from the TCGA; (**B**) Heat map showing methylations of the 4 CpGs in 265 ccRCCs and 133 adjacent normal tissues. Red/blue gradient represents beta values of the methylation sites; (**C**) The ROC curve for the 4 CpGs combination that differentiates between 265 ccRCCs and 133 adjacent normal tissues; (**D**) The difference methylated levels of the 4 CpGs in ccRCC patients with stage I+II and stage III+IV; (**E**) The difference methylated levels of the 4 CpGs in ccRCC patients with tumor grade stage I+II and stage III+IV; (**F**) The percent levels of cg08995609 methylation in 19 ccRCCs and matched adjacent tissues.

To identify a set of CpGs that could best distinguish ccRCCs from adjacent tissues, based on the 986 differential CpGs from promoter regions, we performed shrunken centroids classifier analysis and found 4 hypomethylated CpGs in ccRCC (cg11201447, cg25247520, cg13309012 and cg08995609) that best discriminate between tumor tissues and adjacent tissues. In TCGA specimens (265 ccRCC tissues and 133 adjacent tissues), the 4 CpGs correctly identified 98.113% of the ccRCCs and 98.496% of the adjacent tissues (Fig. 2A). The cg11201447 and cg25247520 remain located in the same transcript of microRNA1204 and had higher degrees of similar methylated levels than other two CpGs, suggesting that methylation levels could be related to chromosomal locations (Fig. 2B). We conducted response operating characteristic (ROC) analysis to evaluate the use of the 4 identified CpGs as a potential biomarker for ccRCC, and obtained a ROC curve with an AUC of 0.997 (95% confidence interval, CI = 0.992–1.000, *P = *4.389E-59) (Fig. 2C). The 4 CpGs methylation in patiens with ccRCC at advanced stages (stage III - IV) were statistically lower than those at early stages (stages I-II) (*P* < 0.05). Comparison with high-grade (grade I-II) showed that the methylation of 3 of 4 CpGs were statistically hypomethylation in low-grade (grade III-IV) ccRCCs (*P* < 0.05), except fot cg08995609, indicating that the 4 CpGs in tumor genesis and progression are dynamic change (Fig. 2D,E).

To validate the possible diagnostic ability of the 4 CpGs, we first performed pyrosequencing analysis of the cg08995609 in RIN1. The methylation level of the cg08995609 in the 19 ccRCCs was significantly hypomethylated (*P* = 0.700E-3) (Fig. 2F). We then analyzed three public datasets, including GSE61441 (46 ccRCCs and matched adjacent tissues from GEO database), E-MTAB-2007 (106 ccRCCs and 6 adjacent tissues from ArrayExpress database) and GSE70303 (6 ccRCCs and matched adjacent tissues from GEO database) that were analyzed in TCGA Illumina HumanMethylation450 microarray. Intriguingly, in all three datasets, cg11201447, cg25247520, cg13309012 and cg08995609 in the ccRCC tissues were significantly lower than in the adjacent tissues (Supplementary Figure 1A). In Supplementary Figure 1B,C,D, ROC curves reveal AUC of 0.996, 0.998 and 1.000 (*P* < 0.05), which are almost identical to that of the TCGA data.

The fact that outstanding biomarkers have tissue specificity prompted us to evaluate methylated levels of the cg11201447, cg25247520, cg13309012 and cg08995609 in other carcinomas. 11 types of cancers were included in the analysis, because the data of these cancers came from more than 10 tumor and adjacent samples, which were detected by Illumina HumanMethylation450 array, and had not suffered from another carcinomas and neoadjuvant therapy. The simultaneous hypomethylations of cg11201447, cg25247520, cg13309012 and cg08995609 with *P* < 0.05 and Delta Beta >  = −0.2 only showed in kidney renal papillary cell carcinoma, but not in the other 10 carcinomas (Supplementary Table 4).

### The correlation of promoter DNA methylation to RAB25, CA9, CYP4B1 and their mRNA expressions

**Figure 3:**
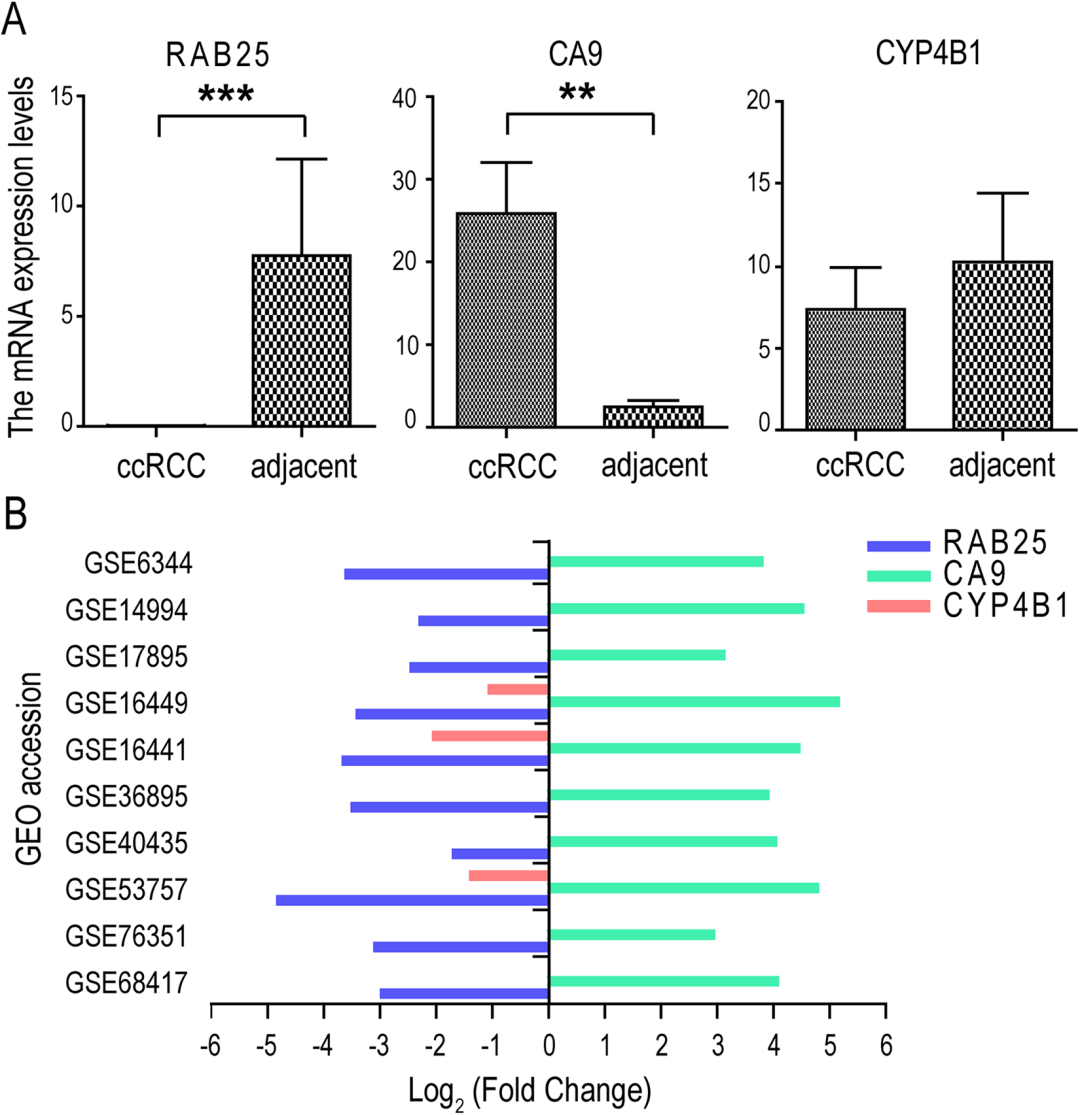
The mRNA expressed levels of RAB25, CA9 and CYP4B1. (**A**) Histogram plot showing mRNA expressed levels of the three genes in 19 ccRCCs and matched adjacent tissues; (**B**) Histogram plot showing differentially expressed levels of the three genes in ten GEO datasets.

From our TCGA data analysis, we found that in ccRCC samples, RAB25 (Delta Beta = 0.202, Methylation *FDR* = 1.080E-56, mRNA *FDR* = 1.180E-38, log2 FC = −3.505) and CYP4B1 (Delta Beta = 0.314, Methylation *FDR* = 1.600E-48, mRNA *FDR* = 4.070E-05, log2 FC = −1.836) are both downregulated with promoter hypermethylations and CA9 is upregulated with promoter hypomethylation (Delta Beta = 0.220, Methylation *FDR* = 6.790E-61, mRNA *FDR* = 2.350E-211, log2 FC = 5.836). As far as we are aware, RAB25 and CYP4B1 have been reported to be related to carcinomas, but it is not clear how the promoter methylations and mRNA expressions of RAB25 and CYP4B1 behave in ccRCC^15,16^. Likewise, our analysis revealed that CYP4B1 was the most differentially hypermethylated gene. The gene CA9 is of particular interest because it has been frequently shown to have diagnostic value in identifying ccRCC, and it is highly expressed through its promoter hypomethylation status in ccRCC^17^. Here, we used CA9 to further check the 19 ccRCC samples. To confirm the differential gene expressions for the three genes, we first used the RNAs from the 19 ccRCCs and matched adjacent tissues to measure their transcript abundances using the qPCR. We found that RAB25 was downregulated (*P* = 0.400E-3) and CA9 was upregulated (*P* = 1.600E-2) in ccRCC tissues, but no differential expression was detected in CYP4B1 (*P* = 0.856) (Fig. 3A). We next analyzed 10 GEO databases and found that in ccRCC tissues, the downregulation of RAB25 and the upregulation of CA9 are consistently repeated. However, CYP4B1 had significantly low expressions in GSE16449, GSE16441 and GSE53757 (Fig. 3B and Supplementary Table 5). Additionally, three upregulated genes with hypomethylations (ESM1, EVI2B, TNFAIP6) and two downregulated genes with hypermethylations (SLC34A1, SOSTDC1) were also confirmed in the 10 GEO datasets (Supplementary Table 5). Among these, ESM1 and TNFAIP6 have been found to be markedly overexpressed in ccRCC, while SOSTDC1 reduced^18–20^.

**Figure 4:**
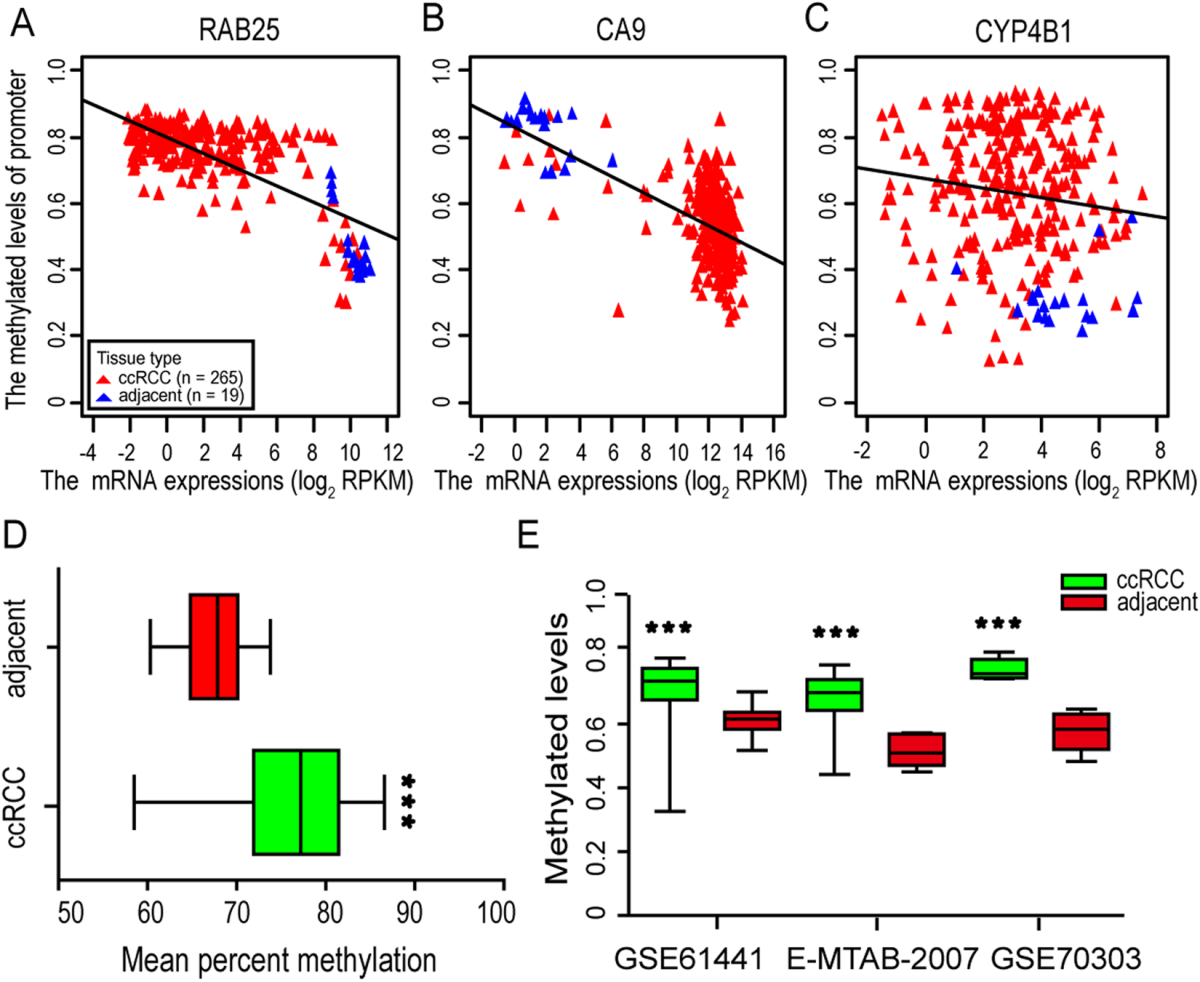
The correlation of promoter methylated levels and mRNA levels and RAB25 promoter methylated levels. (**A**) The correlation of promoter methylated levels and mRNA levels for RAB25. Red triangles denote 265 TCGA ccRCCs, blue triangles denote 19 TCGA adjacent normal tissues; (**B**) The correlation of promoter methylated levels and mRNA levels for CA9; (**C**) The correlation of promoter methylated levels and mRNA levels for CYP4B1. (**D**) Mean percent methylations of RAB25 promoter region in 19 ccRCCs and 19 matched adjacent tissues. (**E**) RAB25 promoter methylated levels in GSE61441, E-MTAB-2007 and GSE70303 datasets.

We further assessed the correlations of promoter DNA methylations with RAB25, CA9, CYP4B1 and their mRNA expressions using Spearman’s rank correlation coefficients. When the mRNA levels of these three genes were plotted against the promoter methylated levels, inverse changes were evident (Fig. 4A,B,C, Spearman: *P* < 0.001, rho = −0.494 for RAB25; *P* < 0.001, rho = −0.538 for CA9; and *P* = 0.020, rho = −0.141 for CYP4B1).

The above analyses prompted us to test the pattern of RAB25 promoter methylation in ccRCC, and we performed MassArray quantitative DNA methylation sequencing of the promoter regions (−918 to −430 bp upstream from the transcription start sites based on UCSC hg19). We found that the RAB25 promoter was significantly hypermethylated (*P* = 0.0003) in 19 ccRCCs (Mean percent methylation = 76.036%) in comparison to the matched adjacent samples (Mean percent methylation = 67.380%) (Fig. 4D). To further validate the relevance of promoter methylation for the RAB25 expression, we compared the percentages of the methylations with qPCR mRNA expressions in the 19 ccRCCs and matched adjacent samples. Once more, significant inverse correlation was found (rho = −0.395, *P* = 0.0141). We also validated the hypermethylation of RAB25 promoter in GSE61441, E-MTAB-2007 and GSE70303 (Fig. 4E). All these are consistent with our TCGA analysis results and suggest that RAB25 promoter hypermethylation may result in gene silencing.

### Methylation levels of RAB25 and CA9, and patients’ survival

Follow-up information on survival was available for the 265 TCGA patients with ccRCC, and by the end of follow-up, 84 patients died. Thus, the median levels of RAB25 and CA9 promoter methylations were cut off, respectively. Kaplan-Meier survival curve stratified by high and low levels of RAB25 promoter methylation was shown in Supplementary Figure 2A. Log-rank test showed that RAB25 promoter methylated levels have no significant association with an unfavorable prognosis for ccRCC patients (*P* = 0.192, Hazard Ratio = 0.752, 95% CI of ratio = 0.491–1.154). As seen in Supplementary Figure 2B, ccRCC patients with low methylated levels of CA9 promoter survived longer than those with high methylated levels of CA9 promoter (*P* = 0.018, Hazard Ratio = 0.594, 95% CI of ratio = 0.385–0.915). We performed Cox proportional hazard regression analysis to determine whether CA9 promoter methylation can be an independent prognostic factor, since univariate analysis indicated that it is a prognostic factor of ccRCC (*P = *0.020) (Table 2). Under the control of potential confounding factors including age, tumor stage, metastasis stage and fuhrman grade, CA9 promoter methylation would not be an independent prognostic variable (*P* = 0.238).

**Table 2:** Univariate and multivariate analysis of prognostic variables for cancer-specific survival in patients with ccRCC.

Variable	No. of patients	Univariate analysis		Multivariate analysis	
		*P*	Hazard ratio (95% CI)	*P*	Hazard ratio (95% CI)
*Age*					
>60	136				
< = 60	128	0.003	1.981(1.260–3.114)	0.007	1.865(1.185–2.938)
*Sex*					
female	92				
male	172	0.536	1.149(0.741–1.781)	—	—
*Tumor stage*					
< = pT2	158				
> = pT3	106	<0.001	3.881(2.419–6.226)	<0.001	2.766(1.706–4.482)
*Node stage*					
pN0	113				
pN1	7				
pNX	144	0.290	0.889(0.715–1.105)	—	—
*Metastasis stage*					
pM0	196				
pM1	46				
pMX	20	<0.001	1.809(1.382–2.368)	<0.001	1.916(1.414–2.597)
*Fuhrman grade*					
< = G2	112				
> = G3	148				
GX	2	<0.001	3.031(1.874–4.900)	0.001	2.589(1.464–4.580)
*CA9 methylation level*					
Low	132				
High	132	0.020	1.704(1.089–2.665)	0.238	1.322(0.831–2.103)

Abbreviation: No., Number.

### GO biological processes and KEGG pathways

To identify biological processes and pathways that enriched by differentially methylated sites within promoters, we analyzed GO and KEGG pathways through the genes that involved in the 986 CpGs. The genes involved in hypomethylated CpGs showed enrichments in the pathways of type I diabetes mellitus (*Bonferroni* = 0.012), cytokine-cytokine receptor interaction (*Bonferroni = *0.017), graft-versus-host disease (*Bonferroni* = 0.026) and the biological processes of immune response and inflammatory response, etc (Supplementary Figure 3A). The genes involved in hypermethylated CpGs, however, were mainly enriched in the pathway of neuroactive ligand-receptor interaction (*Bonferroni* = 4.400E-06), biological processes of cell adhesion, and signaling transduction, etc (Supplementary Figure 3B).

## Discussion

The present study examined promoter methylation changes in tumor and adjacent normal tissues from ccRCC patients. A CpG-based biomarker, including 4 hypomethylated CpG sites (cg11201447, g25247520, cg13309012 and cg0899560) were identified to discriminate ccRCC from adjacent tissues. The validity of the result was further confirmed by pyrosequencing in an independent cohort, and were also observed in other three DNA methylation profiling. The biomarker was reproducible regardless of clinical centre, country or race and it have high sensitivity and specificity. Likewise, the 4 CpGs were found to be more hypomethylated in the late stage than in the early stage of ccRCC, indicating that they could assess the outcome of ccRCC. Distinct heterogeneity across DNA methylation between tumor and adjacent normal tumor tissues provides an opportunity to identify predictive biomarkers of cancers^21,22^, suggesting that the differential CpGs could distinguish malignant tissue from normal tissue. Thus, in a significant fraction of renal biopsies, insufficient tissue is obtained for conventional histologic diagnosis of malignancy and a genomic assay incorporating the 4 CpGs might be useful to improve the diagnose of the individual patient with ccRCC.

Ting *et al*. reported that DNA methylation altered pattern is globally demethylated^6^. Mantle cell lymphoma has a surprising outstanding gene promoter hypomethylation^23^. In early stages of carcinogenesis, the appearing of DNA hypomethylation seems to cause genomic instability and activate the transcriptions and expressions of proto-oncogenes, which may contribute to cancer pathogenesis^24^. Thus, we can assume that promoter hypomethylation preferentially contribute to ccRCC pathologies. There are two microRNAs and a gene correspond to the 4 identified CpGs: microRNA-1204, microRNA-155 and RIN1. Human microRNA-1204, which is residing in 60 kb downstream of MYC and within the exon 1b of PVT1, depresses tumor suppressor genes and contributes to ovarian and breast tumor proliferations and patients’ survival^25^. An increased expression of miR-1204 was suggested to play a role in the development of B cell malignancy and could influence the cell proliferations and migrations of prostate and breast tumors^26^. It has been reported that microRNA-155 involved in the tumorigenesis of human breast and colorectal^27,28^. Compared with normal kidney tissue, microRNA-155 had a significant overexpression in ccRCC, so it could serve as a predictive marker for survival in patients with stage III and IV ccRCC^29^. MicroRNA-155 could promote cell proliferations and migratory activities and it could suppress apoptosis in renal cancer cell by targeting a suppressor gene BACH1^30^. As an ABL tyrosine kinase activator and an RAS effector, RIN1 was connected with many key regulatory mechanisms. It had been described as an oncogene in human non-small cell lung adenocarcinoma and bladder urothelial^31,32^. However, for breast cancer, DNA methylation with RIN1 promoter was involved in silencing its expression, which may contribute to adenocarcinoma progression^33^. It has been reported that the cg14391855 located at RIN1 was one of prognostic and diagnostic markers for ccRCC by Wei *et al*. and Lasseigne *et al*., respectively^21,34^. Our TCGA study revealed that RIN1 promoter in ccRCC tissues was hypomethylated (*FDR* = 9.850E-76, |Delta Beta| = −0.251), suggesting that DNA methylation of RIN1 promoter could be related to the ccRCC. Our study suggested that tumorous epigenetic marker drifts could occur preferentially in transcribed regions, which is an interesting insight in neoplasm.

Oncogenes and tumor suppressor genes within promoter regions induce aberrant DNA methylation, resulting in the development and progression of neoplasms^12^. Our goal of this study is to detect tumor-related genes that suffered from methylation variations within promoter regions. It was reported that the tyrosine of CA9 could be phosphorylated in an epidermal growth factor dependent manner, interacts with the regulatory subunit of PI-3-Kinase, and activates Akt^35^. There does not seem to be any report on the relationship between CA9 promoter methylation and ccRCC patients’ survival, though there exist many reports on CA9 expression as a predictive maker of ccRCC^36^. Our analysis of TCGA data showed that higher levels of CA9 promoter methylation can accelerate ccRCC patients’ death and it could be a predictor of outcome in patients with ccRCC, but its reliability is limited as an independent predictor. Two published studies have reported that CA9 would not an independent predictor of outcomes for patients with ccRCC^37,38^. Numerous studies have shown that RAB25, a rab superfamily protein of small GTPases, acts as a tumor suppressor or an oncogene in the tumorigenicity of several types of cancers^18,39,40^. The linkage between the depletion of RAB25 and promoter hypermethylation in esophageal squamous cell carcinoma has been reported^18^. Our analysis showed that in comparison with the adjacent tissues, RAB25 in ccRCC had a significant increase in methylation and a lower level of mRNA expression. Thus, the inactivation of RAB25 caused by promoter hypermethylation could have considerable effects on the development and progression of ccRCC. However, Liu *et al*. showed that RAB25 protein expression was upregulated in ccRCC tissues relative to paired adjacent noncancerous tissues and that it was significantly correlated with the tumorous advance stages^41^. This discordance in mRNA and protein levels of RAB25 expressions may be interpreted by posttranscriptional control mechanisms, such as upstream open reading frames, ironresponsive elements, and microRNAs^42,43^. In our study, the levels of CYP4B1 mRNA using qPCR were evaluated in the 19 ccRCCs, but we did not notice a significantly difference. Our analysis showed that only 3 of the 10 GEO datasets showed that CYP4B1 mRNA levels were upregulated in ccRCC, which is consistent with the analysis of TCGA data. The inconsistency between our analysis and the published results could be the result of the fact that different technologies and origins of samplings can lead to sensitivities in the detections of mRNA levels. Our Spearman’s rank correlation analysis indicated that RAB25 and CA9 promoter methylations are likely to be inversely involved in regulating their mRNA expressions and thus they participate in the progressions of ccRCC.

The combination of differentially expressed CpGs and biological regulatory mechanisms in ccRCC showed decreased methylation enrichments in the immune and inflammatory response. Similar findings have been reported by Brittany N Lasseigne *et al*.^21^. The fact that the genes implicated in the immune and inflammatory responses of ccRCC are upregulated could be explained by DNA methylation changes^44^. Three hypomethylated pathways consisting of type I diabetes mellitus, cytokine-cytokine receptor interaction and graft-versus-host disease, were interpreted as upregulated pathways involved in ccRCC^45^. It has been known that cell-cell adhesion are important in the formation of histogenesis and cell societies^46^. The mutual adhesiveness of normal cells is significantly stronger than those of the cancerous ones^47^. Our study also showed that increased methylation are associated with cell adhesion-related and signaling-related biological processes. The CpG hypermethylation of the promoter region of the E-cadherin gene mediated cell adhesions and inactivated its mRNA expression in renal cell carcinoma^48^. Therefore, promoter hypermethylation depresses cell adhesion-related genes’ expressions, which contribute to the malignance of ccRCC cells. These suggest that, indirectly, promoter DNA methylation could regulate critical genes of biological processes or pathways, and thus alter the effects of processes or pathways in ccRCC progression.

By approaching DNA methylation in cancer from a genomic perspective, we were able to gain new insights into the underlying biology of ccRCC, as well as found a novel CpG-based biomarker for the diagnosis of the disease. However, our study was partly limited, our cohort was clinically representative of patients presenting with the disease, the absence of methylation detection of cancer-free individuals limits our conclusion. If robust diagnostic markers can be identified, they will need to be developed into biomarkers for use in a clinical setting. Therefore, the biomarker need to be validated by large-scale clinical trials, ideally prospectively, to finalize the convincing specificity and sensitivity. Meanwhile, our findings of aberrant DNA methylation in ccRCC need to be more explored in the vitro and vivo model.

In conclusion, we have showed that together the methylation levels of cg11201447, cg25247520, cg13309012 and cg08995609 CpGs could be a potential biomarker for ccRCC. The hypomethylated RAB25 promoter leading to its downregulation may promote the ccRCC carcinogenesis.

## Methods

### The Cancer Genome Atlas data

All ccRCC patients’ data were from TCGA data portal up to August 29, 2015. The data of the patients who have have suffered from other malignancies, or received neoadjuvant therapy, or no evaluable ethnicity, were first removed. Then full clinical data, methylation values (level 1 data, Illumina Infinium HumanMethylation 450 K) and mRNA expressions (level 3 data, RNA-seq Version 2) were downloaded. Adjacent tissues had a distance from the tumor margin greater than 2 cm.

### Illumina Infinium HumanMethylation 450 K analysis

We analyzed TCGA Illumina HumanMethylation450 array of ccRCC using RnBeads version 0.99.19 in the R software 3.1.2, where the methylation signal data was extracted and processed. In processing and filtering, we filtered out a probe when the last 5 bases in its target sequence overlap with SNP and removed CpG sites with more than 10% missing values in all samples. Methylation measures with a detection P-value > 0.01 were removed. Both sites and samples were filtered using a greedy approach. In addition, CpG sites on the sex chromosomes were removed to avoid sex-specific methylation bias. Background subtraction with method “methylumi.noob” and normalization with method “swan” were performed respectively. Surrogate variable analysis (SVA) was performed to exclude covariates against the variable of tissue types (tumor and adjacent). Limma package was used to identify differentially methylated CpGs and genes between ccRCC and adjacent tissues. In the analysis, the reference human genome (hg19) was used to annotate all genes and CpGs.

### The 19 ccRCC tissue samples that were used for validation

The 19 ccRCC and matched adjacent tissues used for validation were collected at the Anhui provincial hospital between 2011 and 2012 with patients’ informed consents. They were obtained from surgical resections, freshfrozen, and stored at −80 °C. The 19 ccRCCs were confirmed without other medical history or receiving chemotherapy or radiotherapy. The tumors were graded according to the 2010 AJCC cancer staging manual and the future of TNM. Detailed clinical information was provided in Supplementary Table 6.

### Quantitative DNA Methylation

Genomic DNAs from the 19 ccRCC tissues were extracted with TaKaRa MiniBEST Universal Genomic DNA Extraction Kit Ver.5.0. DNA concentrations were measured with a NanoDrop2000 spectrophotometer (USA). The methylation levels of CpG sites were evaluated with pyrosequencing. PyroMark Assay Design software (Qiagen) was used to design specific sets of primers for CpG PCR amplification and sequencing. The cg08995609 forward primer: GAGGGTTTTAGTTGGGGGATGTTA, reverse primer: ACCTAAAACCAAAAACAAATAAACAACT, sequencing primer: GTTGGGGGATGTTAT. All manipulations of bisulfite conversions, PCRs and pyrosequencings were previously described^33^. DNA methylation of RAB25 promoter was quantified by MassARRAY EpiTYPER assays (Sequenom, San Diego, CA, USA). Primers were designed using the software sequenom®EpiDesigner, RAB25 sequences of PCR primers used in the study, tag-forward: aggaagagagGTATTGTTGGGTTTTTGGAATTTGT; tag-reverse: cagtaatacgactcactatagggagaaggctATCTCAACCCCTAAAACCTCTACC. Procedures of methylation assessments and quality controls had been described previously^49^.

### Real-time PCR analysis

Total RNAs of the 19 ccRCC tissues were extracted using the Axygen RNA Isolation Kit (USA) according to the manufacturer’s instructions. RNA concentrations were measured with a NanoDrop2000 spectrophotometer (USA). cDNA was synthesized from 1ug of total RNA using PrimeScript® 1st strand cDNA Synthesis Kit (TaKaRa, China) according to the manufacturer’s protocol. Real-time polymerase chain reactions to assess the mRNA expression levels of RAB25, CA9 and CYP4B1 were carried out using SYBR® Premix Ex Taq™ II (TaKaRa, China) with a 7500 Real-Time PCR system (Applied Biosystems, CA, USA). Glyceraldehyde 3-phosphate dehydrogenase (GAPDH) was used as an internal control gene. The primer sequences used in the study were: GAPDH: forward primer-GCACCGTCAAGGCTGAGAAC, reverse primer-TGGTGAAGACGCCAGTGGA; CA9: forward primer-GGCTGCTGGTGACATCCTA, reverse primer-AGTCTCGGCTACCTCTGCTG; RAB25: forward primer-TCGCTGAAAACAATGGACTG, reverse primer -CGGATGCTG TTCTGTCTCTG; CYP4B1: forward primer-TCTACTGGCTCACCCCACAT, reverse primer-TTCTTCCGCACCTTCTCATC. Relative expression values were calculated following the 2^−ΔΔCt^ method^50^.

### Gene Expression Omnibus (GEO) datasets

The information on Illumina HumanMethylation450 array studies for ccRCC was systematically searched using the search string (Renal clear cell carcinoma and methylation), the information on mRNA expression profile or sequencing for ccRCC was obtained by searching the string (Renal clear cell carcinoma and (mRNA OR gene)). The search was restricted to the datasets published up to February 1, 2016. Our selection criteria were: (a) The original experimental study provided a comparison of ccRCC tissues and non-tumor tissues; (b) the studied samples did not receive neoadjuvant therapy; (c) the studied organisms were Homo sapiens. The datasets, including GSE68417, GSE76351, GSE53757, GSE40435, GSE36895, GSE16441, GSE16449, GSE17895, GSE14994, GSE6344, were applied to validate the mRNA expressions.

### Statistical analysis

To identify diagnostic markers, shrunken centroids classifier analysis was performed on differential CpGs within promoter using PamR (version 1.55, see manual in R). Based on visual examination of the training errors and cross-validation results, we optimally minimized the miss-rate and set the shrinkage threshold to 18.00 for ccRCC and adjacent tissue classification. Based on RNA-seq raw read counts, differential mRNA expression analysis was implemented by the DESeq Bioconductor package (version 1.18.0) in R. To analyze the biological functions that are regulated by DNA methylation, we first linked differential CpGs with the located genes, and then analyzed these genes for Gene Ontology and KEGG PATHWAY based on the DAVID database. Wilcoxon’s matched pairs sign rank tests for paired data and spearman’s rank-correlation coefficients were performed in GraphPad Prism 5.0 software. Log-rank test and cox’s proportional hazards regression model were calculated in IBM SPSS Statistics Version 22.0, in which, the predicted values from the logistic regression model were used to construct receiver operating characteristic (ROC) curves and then to calculate the area under the ROC curve (AUC). *P* values less than 0.05 were considered statistically significant.

### Informed Consent

Written informed consent was obtained from each participant prior to tumor samples collection. All of the clinical samples were obtained from Anhui Provincial Hospital (Anhui, China). The study protocol was approved by the Clinical Research Ethics Committee of Anhui Provincial Hospital.

## Electronic supplementary material

Supplementary Information

Dataset 1

Dataset 2

Dataset 3

Dataset 4

Dataset 5

Dataset 6
