# Supplementary material for: Promoter DNA methylation analysis reveals a novel diagnostic CpG-based biomarker and RAB25 hypermethylation in clear cell renel cell carcinoma
Source: Sci Rep. 2017 Oct 27;7:14200. doi: 10.1038/s41598-017-14314-y (PMC5660223; doi:10.1038/s41598-017-14314-y)
Supplement: Supplementary file 1 — Supplementary Information [file 41598_2017_14314_MOESM1_ESM.doc]

**Promoter DNA methylation analysis reveals** **a novel** **diagnostic** **CpG-based biomarker and RAB25 hypermethylation in clear cell renel cell carcinoma**

Yinmin Gu^1§^ Yi Ming Zou^2§^ Danqing Lei^1§^ Yuanjie Huang^1^ Weidong Li^1^ Zengnan Mo^3^ Yanling Hu^1,3,4^*

1 Life Sciences Institute, Guangxi Medical University, Nanning, Guangxi, 530021, China

2 Department of Mathematical Sciences, University of Wisconsin-Milwaukee, Milwaukee, WI, 53201, USA

3 Center for Genomic and Personalized Medicine, Guangxi Medical University, Nanning, Guangxi, 530021, China

4 Guangxi Colleges and Universities Key Laboratory of Biological Molecular Medicine Research, Guangxi Medical University, Nanning, Guangxi, 530021, China

* Correspondence: huyanling@gxmu.edu.cn

^§^ These authors contributed equally.

Supplementary Table 1. 986 differential CpGs within promoter between 265 ccRCC and 133 adjacent tissues.

Supplementary Table 2. List of the 173 differentially methylated genes within promoter.

Supplementary Table 3. 4438 differential expression genes between 525 ccRCC and 69 adjacent tissues.

Supplementary Table 4. Methylation conditions of cg11201447, cg25247520, cg13309012 and cg08995609 in eleven cancer types.

Supplementary Table 5. The mRNA expressions of genes with differential promoter methylations and mRNA regulations in 10 GEO datasets.

Supplementary Table 6. Characteristics of 19 ccRCC samples from the Anhui provincial hospital.


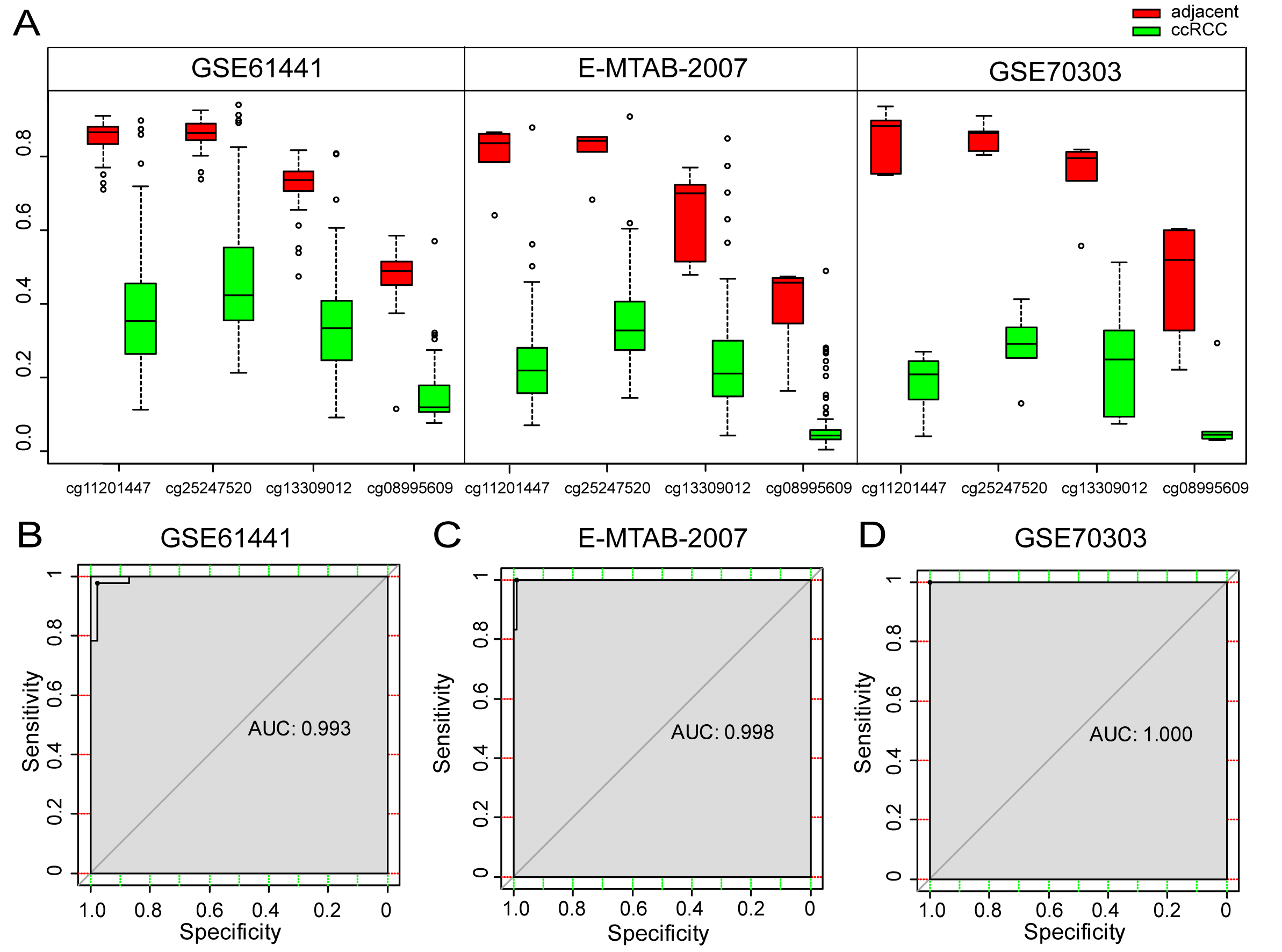


Supplementary Figure 1. Validations of the 4 CpGs methylated levels and their combined diagnostic performance.


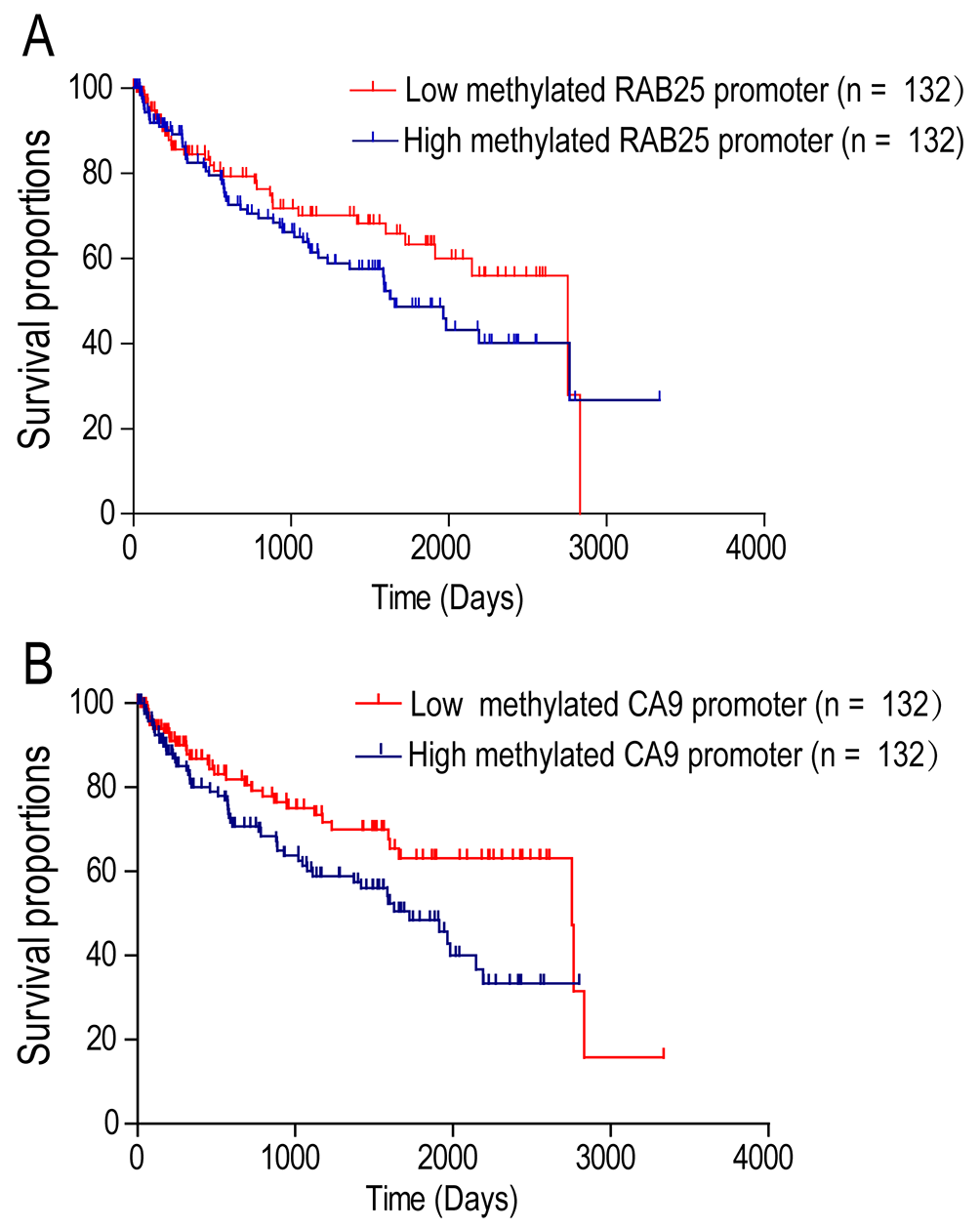


Supplementary Figure 2. Kaplan-Meier plot of overall ccRCC survival.


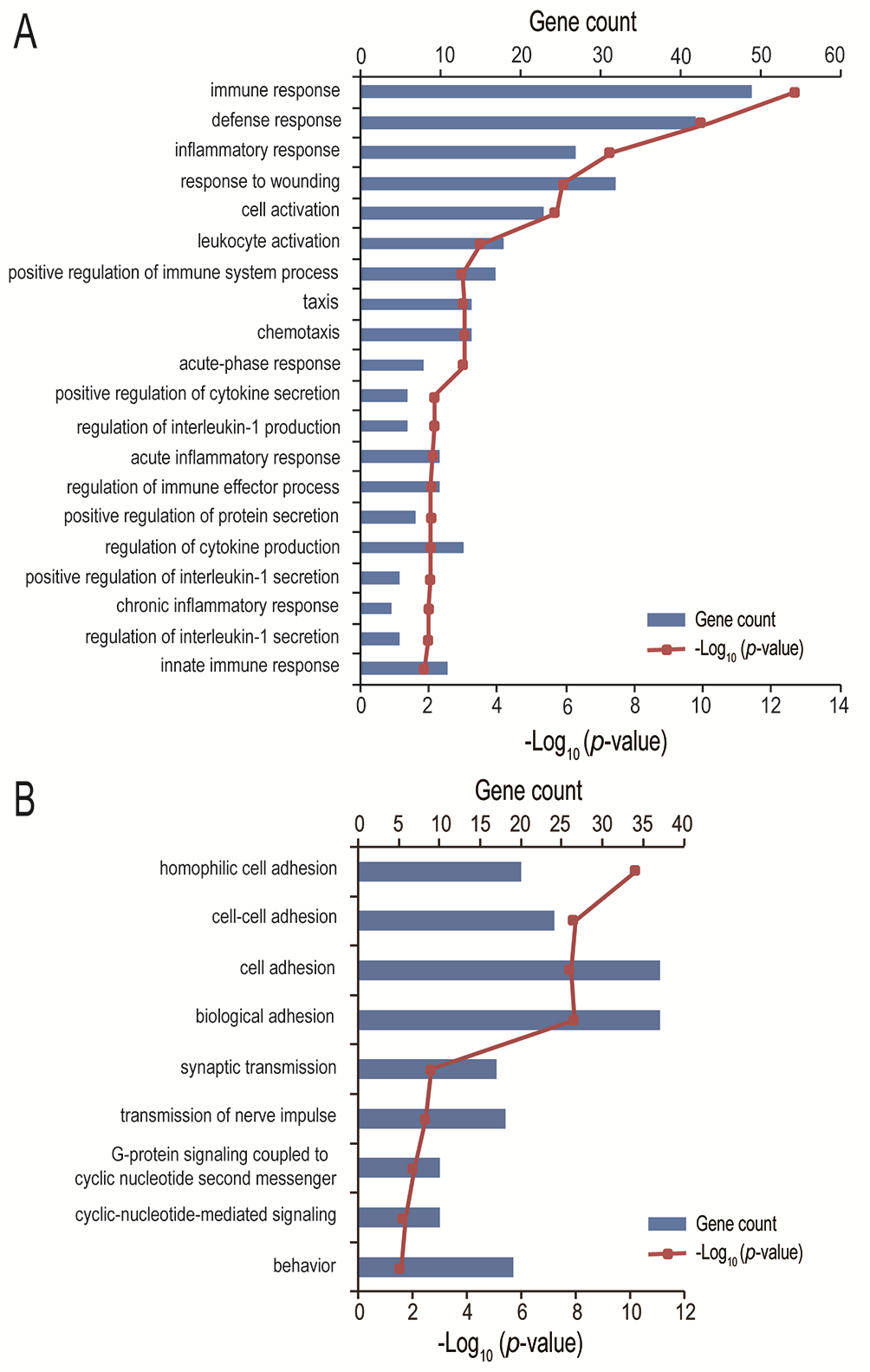


Supplementary Figure 3. Bar graph showing biological processes of genes involving differential methylated CpGs.
