# Supplementary material for: Promoter DNA methylation analysis reveals a novel diagnostic CpG-based biomarker and RAB25 hypermethylation in clear cell renel cell carcinoma
Source: Sci Rep. 2017 Oct 27;7:14200. doi: 10.1038/s41598-017-14314-y (PMC5660223; doi:10.1038/s41598-017-14314-y)
Supplement: Supplementary file 3 — Dataset 2 [file 41598_2017_14314_MOESM3_ESM.doc]

Table S2. List of the 173 differentially methylated genes within promoter

| Chromosome | Start | End | | Symbol | | Delta Beta | | *FDR* | |
| --- | --- | --- | --- | --- | --- | --- | --- | --- | --- |
| 4 | 74604723 | 74606722 | | IL8 | | -0.427 | | 1.48E-164 | |
| 11 | 62323208 | 62325207 | | AHNAK | | -0.400 | | 1.24E-89 | |
| 11 | 43941995 | 43943994 | | ALKBH3-AS1 | | -0.392 | | 2.04E-87 | |
| 13 | 37064079 | 37066078 | | HIST1H2APS6 | | -0.362 | | 1.64E-78 | |
| 12 | 127256458 | 127258457 | | LINC00944 | | -0.362 | | 8.48E-80 | |
| 13 | 25315315 | 25317314 | | IRX1P1 | | -0.359 | | 2.38E-62 | |
| 2 | 225874758 | 225876757 | | MIR4439 | | -0.356 | | 2.71E-86 | |
| 6 | 76781896 | 76783895 | | IMPG1 | | -0.352 | | 8.04E-72 | |
| 14 | 51794605 | 51796604 | | LINC00519 | | -0.342 | | 1.11E-83 | |
| 20 | 43834138 | 43836137 | | SEMG1 | | -0.339 | | 3.41E-71 | |
| 5 | 32530239 | 32532238 | | SUB1 | | -0.335 | | 1.38E-56 | |
| 6 | 149805698 | 149807697 | | ZC3H12D | | -0.332 | | 4.31E-101 | |
| 1 | 182375256 | 182377255 | | LINC00272 | | -0.328 | | 1.35E-83 | |
| 17 | 46179219 | 46181218 | | SNX11 | | -0.326 | | 1.28E-42 | |
| 10 | 43835734 | 43837733 | | RNU6ATAC11P | | -0.326 | | 1.45E-75 | |
| 12 | 31900532 | 31902531 | | STMN1P1 | | -0.322 | | 6.86E-44 | |
| 12 | 116995686 | 116997685 | | MAP1LC3B2 | | -0.319 | | 8.35E-86 | |
| 10 | 14816397 | 14818396 | | FAM107B | | -0.316 | | 2.07E-79 | |
| 12 | 52604140 | 52606139 | | C12orf80 | | -0.316 | | 3.4E-57 | |
| 17 | 57917127 | 57919126 | | MIR21 | | -0.303 | | 5.34E-94 | |
| 16 | 82225896 | 82227895 | | RN7SKP190 | | -0.300 | | 9.93E-50 | |
| 1 | 158655989 | 158657988 | | SPTA1 | | -0.292 | | 5.97E-40 | |
| 2 | 120996140 | 120998139 | | RALB | | -0.291 | | 1.07E-51 | |
| 22 | 30877399 | 30879398 | | SDC4P | | -0.291 | | 3.18E-80 | |
| 7 | 38402620 | 38404619 | | TRGV2 | | -0.286 | | 1.97E-71 | |
| 11 | 63272056 | 63274055 | | LGALS12 | | -0.285 | | 1.71E-69 | |
| 8 | 134313766 | 134315765 | | NDRG1 | | -0.280 | | 9.09E-47 | |
| 2 | 139653221 | 139655220 | | YY1P2 | | -0.280 | | 4.78E-66 | |
| 2 | 87753387 | 87755386 | | LINC00152 | | -0.275 | | 8.83E-79 | |
| 22 | 38029161 | 38031160 | | SH3BP1 | | -0.275 | | 2.51E-36 | |
| 1 | 25348494 | 25350493 | | MIR4425 | | -0.274 | | 2.37E-48 | |
| 8 | 57471883 | 57473882 | | LINC00968 | | -0.270 | | 5.5E-49 | |
| 5 | 76144326 | 76146325 | | S100Z | | -0.269 | | 4.82E-51 | |
| 3 | 57092969 | 57094968 | | SPATA12 | | -0.268 | | 2.24E-62 | |
| 17 | 69293860 | 69295859 | | RNU7-155P | | -0.266 | | 6.58E-74 | |
| 19 | 17515958 | 17517957 | | BST2 | | -0.265 | | 1.65E-72 | |
| 19 | 17515031 | 17517030 | | MVB12A | | -0.265 | | 1.65E-72 | |
| 5 | 147646243 | 147648242 | | SPINK13 | | -0.263 | | 1.87E-51 | |
| 3 | 45957035 | 45959034 | | LZTFL1 | | -0.260 | | 2.73E-64 | |
| 14 | 81425362 | 81427361 | | CEP128 | | -0.255 | | 3.55E-43 | |
| 15 | 42719396 | 42721395 | | RNU6-188P | | -0.253 | | 8.01E-75 | |
| Chromosome | Start | | End | | Symbol | | Delta Beta | | *FDR* |
| 11 | 124118923 | | 124120922 | | OR8G1 | | -0.252 | | 5.98E-103 |
| 11 | 120105849 | | 120107848 | | POU2F3 | | -0.252 | | 2.26E-58 |
| 11 | 66103812 | | 66105811 | | RIN1 | | -0.251 | | 9.85E-76 |
| 7 | 45018198 | | 45020197 | | MYO1G | | -0.250 | | 1.75E-66 |
| 17 | 7481285 | | 7483284 | | CD68 | | -0.249 | | 2.54E-72 |
| 1 | 1711397 | | 1713396 | | NADK | | -0.249 | | 5.38E-42 |
| 1 | 153122846 | | 153124845 | | SPRR2G | | -0.249 | | 6.97E-50 |
| 5 | 58823187 | | 58825186 | | NDUFB4P2 | | -0.245 | | 4.91E-56 |
| 3 | 52864996 | | 52866995 | | ITIH4 | | -0.245 | | 8.96E-71 |
| 14 | 105531283 | | 105533282 | | GPR132 | | -0.242 | | 1.98E-59 |
| 21 | 22175035 | | 22177034 | | LINC00320 | | -0.241 | | 9.29E-36 |
| 1 | 153013908 | | 153015907 | | SPRR2D | | -0.240 | | 1.5E-56 |
| 6 | 31145177 | | 31147176 | | PSORS1C3 | | -0.240 | | 4.78E-64 |
| 19 | 4539987 | | 4541986 | | LRG1 | | -0.239 | | 6.39E-59 |
| 11 | 89608686 | | 89610685 | | TRIM64B | | -0.239 | | 7.12E-55 |
| 2 | 152212606 | | 152214605 | | TNFAIP6 | | -0.239 | | 2.54E-61 |
| 10 | 74396024 | | 74398023 | | HMGN2P34 | | -0.239 | | 1.06E-30 |
| 5 | 90609720 | | 90611719 | | LUCAT1 | | -0.238 | | 1.07E-117 |
| 9 | 104138386 | | 104140385 | | FYTTD1P1 | | -0.237 | | 2.8E-48 |
| 1 | 34324576 | | 34326575 | | HMGB4 | | -0.236 | | 3.38E-52 |
| 11 | 124133340 | | 124135339 | | OR8G5 | | -0.236 | | 1.92E-46 |
| 17 | 42993806 | | 42995805 | | GFAP | | -0.236 | | 8.04E-63 |
| 9 | 5509045 | | 5511044 | | PDCD1LG2 | | -0.235 | | 7.65E-45 |
| 1 | 153002178 | | 153004177 | | SPRR1B | | -0.235 | | 6.33E-64 |
| 12 | 109491258 | | 109493257 | | USP30-AS1 | | -0.235 | | 7.42E-30 |
| 1 | 196787375 | | 196789374 | | CFHR1 | | -0.235 | | 8.62E-49 |
| 1 | 196787398 | | 196789397 | | CFHR2 | | -0.235 | | 8.62E-49 |
| 1 | 204676059 | | 204678058 | | RNA5SP75 | | -0.235 | | 3.27E-49 |
| 5 | 176804736 | | 176806735 | | SLC34A1 | | -0.234 | | 3.72E-36 |
| 5 | 9547448 | | 9549447 | | SNORD123 | | -0.234 | | 2.53E-39 |
| 20 | 43882706 | | 43884705 | | SLPI | | -0.233 | | 4.67E-52 |
| 7 | 142421673 | | 142423672 | | TRBV27 | | -0.233 | | 1.59E-36 |
| 22 | 40295586 | | 40297585 | | GRAP2 | | -0.231 | | 2.58E-56 |
| 8 | 24150053 | | 24152052 | | ADAM28 | | -0.231 | | 1.25E-35 |
| 12 | 10022236 | | 10024235 | | CLEC2B | | -0.230 | | 4.67E-52 |
| 17 | 56357797 | | 56359796 | | MPO | | -0.229 | | 8.6E-77 |
| 5 | 95158210 | | 95160209 | | GLRX | | -0.228 | | 1.36E-50 |
| 1 | 31970026 | | 31972025 | | RNU6-40P | | -0.226 | | 1.28E-52 |
| 20 | 121510 | | 123509 | | DEFB126 | | -0.225 | | 5.63E-36 |
| 10 | 687219 | | 689218 | | MIR5699 | | -0.225 | | 1.79E-43 |
| 3 | 143566874 | | 143568873 | | SLC9A9 | | -0.224 | | 4.96E-39 |
| 19 | 49528778 | | 49530777 | | NTF6A | | -0.224 | | 9.55E-81 |
| Chromosome | Start | | End | | Symbol | | Delta Beta | | *FDR* |
| 1 | 159795040 | | 159797039 | | SLAMF8 | | -0.223 | | 1.83E-52 |
| 6 | 55190767 | | 55192766 | | GFRAL | | -0.222 | | 7.31E-48 |
| 5 | 113587710 | | 113589709 | | RN7SKP89 | | -0.221 | | 2.59E-47 |
| 15 | 89177884 | | 89179883 | | ISG20 | | -0.221 | | 5.68E-33 |
| 9 | 35672353 | | 35674352 | | CA9 | | -0.220 | | 6.79E-61 |
| 20 | 54985668 | | 54987667 | | CASS4 | | -0.219 | | 9.47E-45 |
| 16 | 66636277 | | 66638276 | | CMTM3 | | -0.219 | | 1.53E-28 |
| 11 | 3069252 | | 3071251 | | RNU1-91P | | -0.218 | | 1.26E-71 |
| 1 | 177938849 | | 177940848 | | SEC16B | | -0.217 | | 1.61E-38 |
| 22 | 22597587 | | 22599586 | | VPREB1 | | -0.216 | | 8.22E-47 |
| 7 | 120723368 | | 120725367 | | RNA5SP241 | | -0.216 | | 3.17E-34 |
| 5 | 54318000 | | 54319999 | | ESM1 | | -0.216 | | 1.01E-27 |
| 5 | 54318581 | | 54320580 | | GZMK | | -0.216 | | 1.01E-27 |
| 11 | 124142366 | | 124144365 | | SLC5A4P1 | | -0.215 | | 1.67E-23 |
| 3 | 189839727 | | 189841726 | | LEPREL1 | | -0.213 | | 5.97E-57 |
| 1 | 152669340 | | 152671339 | | LCE2A | | -0.212 | | 6.51E-47 |
| 17 | 47785877 | | 47787876 | | SLC35B1 | | -0.212 | | 1.46E-36 |
| 1 | 68840847 | | 68842846 | | TCEB1P18 | | -0.211 | | 1.23E-34 |
| 5 | 70320442 | | 70322441 | | NAIP | | -0.211 | | 6.37E-36 |
| 10 | 10836444 | | 10838443 | | SFTA1P | | -0.211 | | 9.01E-35 |
| 20 | 43802017 | | 43804016 | | PI3 | | -0.211 | | 2.33E-57 |
| 14 | 101401328 | | 101403327 | | SNORD113-4 | | -0.210 | | 1.96E-44 |
| 17 | 72527114 | | 72529113 | | CD300LB | | -0.209 | | 3.32E-58 |
| 3 | 160120876 | | 160122875 | | MIR15B | | -0.209 | | 3.01E-37 |
| 3 | 160121033 | | 160123032 | | MIR16-2 | | -0.209 | | 3.01E-37 |
| 15 | 100015870 | | 100017869 | | MEF2A | | -0.209 | | 5.37E-29 |
| 1 | 157670148 | | 157672147 | | FCRL3 | | -0.208 | | 1.86E-49 |
| 19 | 22379254 | | 22381253 | | ZNF676 | | -0.208 | | 2E-25 |
| 12 | 113414700 | | 113416699 | | OAS2 | | -0.208 | | 2.53E-65 |
| 11 | 55904695 | | 55906694 | | OR8J3 | | -0.208 | | 6.85E-26 |
| 5 | 22140961 | | 22142960 | | PMCHL1 | | -0.207 | | 5.36E-48 |
| 7 | 16569706 | | 16571705 | | SOSTDC1 | | -0.207 | | 1.06E-68 |
| 2 | 134325535 | | 134327534 | | NCKAP5 | | -0.206 | | 5.31E-48 |
| 11 | 124469980 | | 124471979 | | OR8Q1P | | -0.206 | | 3.79E-32 |
| 9 | 125279920 | | 125281919 | | OR1J4 | | -0.206 | | 1.81E-21 |
| 19 | 6591840 | | 6593839 | | RPL7P50 | | -0.206 | | 1.42E-45 |
| 13 | 106807340 | | 106809339 | | RNA5SP38 | | -0.205 | | 2.35E-54 |
| 12 | 47609740 | | 47611739 | | PCED1B-AS1 | | -0.205 | | 9.17E-46 |
| 10 | 103065786 | | 103067785 | | RNU2-43P | | -0.204 | | 1.14E-28 |
| 15 | 58428868 | | 58430867 | | AQP9 | | -0.204 | | 2.22E-57 |
| 5 | 179219481 | | 179221480 | | LTC4S | | -0.204 | | 1.56E-36 |
| 11 | 4936423 | | 4938422 | | OR51G2 | | -0.203 | | 4E-44 |
| Chromosome | Start | | End | | Symbol | | Delta Beta | | *FDR* |
| 19 | 47735524 | | 47737523 | | BBC3 | | -0.203 | | 5.35E-77 |
| 7 | 134232388 | | 134234387 | | AKR1B15 | | -0.203 | | 3.78E-71 |
| 7 | 112727334 | | 112729333 | | GPR85 | | -0.203 | | 8.31E-24 |
| 10 | 43243636 | | 43245635 | | DUXAP3 | | -0.202 | | 6.15E-30 |
| 2 | 219838821 | | 219840820 | | LINC00608 | | -0.202 | | 1.01E-29 |
| 5 | 170292339 | | 170294338 | | RN7SL623P | | -0.201 | | 3.02E-42 |
| 19 | 57270801 | | 57272800 | | OR5AH1P | | -0.201 | | 5.03E-45 |
| 1 | 54199378 | | 54201377 | | GLIS1 | | -0.201 | | 8.95E-51 |
| 11 | 55735491 | | 55737490 | | OR10AG1 | | -0.201 | | 4.17E-34 |
| 14 | 101394756 | | 101396755 | | SNORD113-3 | | -0.200 | | 2.75E-32 |
| 19 | 45115440 | | 45117439 | | IGSF23 | | -0.200 | | 7.88E-18 |
| 17 | 29640631 | | 29642630 | | EVI2B | | -0.200 | | 2.22E-33 |
| 3 | 194030093 | | 194032092 | | LINC00887 | | -0.200 | | 1.48E-63 |
| 2 | 166324657 | | 166326656 | | CSRNP3 | | 0.200 | | 1.54E-47 |
| 10 | 50506564 | | 50508563 | | C10orf71-AS1 | | 0.200 | | 4.67E-52 |
| 19 | 6464715 | | 6466714 | | SLC25A23 | | 0.201 | | 6.02E-31 |
| 19 | 36424760 | | 36426759 | | LRFN3 | | 0.202 | | 4.17E-38 |
| 1 | 156029451 | | 156031450 | | RAB25 | | 0.202 | | 1.08E-56 |
| 7 | 138481195 | | 138483194 | | TMEM213 | | 0.203 | | 2.30E-55 |
| 6 | 26015107 | | 26017106 | | HIST1H1PS2 | | 0.203 | | 2.57E-58 |
| 7 | 27191701 | | 27193700 | | HOXA3 | | 0.203 | | 3.02E-35 |
| 18 | 70534882 | | 70536881 | | NETO1 | | 0.205 | | 2.79E-59 |
| 2 | 132087931 | | 132089930 | | ARHGAP42P1 | | 0.205 | | 2.22E-59 |
| 7 | 24322282 | | 24324281 | | NPY | | 0.205 | | 1.46E-36 |
| 6 | 105387903 | | 105389902 | | LINC00577 | | 0.205 | | 8.06E-53 |
| 19 | 57048817 | | 57050816 | | ZFP28 | | 0.206 | | 1.17E-55 |
| 5 | 140742398 | | 140744397 | | PCDHGA5 | | 0.208 | | 2.84E-71 |
| 7 | 27145896 | | 27147895 | | HOXA-AS2 | | 0.208 | | 7.43E-59 |
| 3 | 39195554 | | 39197553 | | CSRNP1 | | 0.209 | | 5.77E-37 |
| 7 | 27182788 | | 27184787 | | HOXA5 | | 0.210 | | 3.02E-74 |
| 3 | 149685673 | | 149687672 | | ANKUB1 | | 0.210 | | 1.16E-40 |
| 12 | 6482711 | | 6484710 | | LTBR | | 0.212 | | 3.55E-74 |
| 2 | 233251668 | | 233253667 | | ECEL1P2 | | 0.214 | | 3.90E-64 |
| 1 | 65712402 | | 65714401 | | DNAJC6 | | 0.217 | | 1.21E-44 |
| 3 | 53188525 | | 53190524 | | PRKCD | | 0.218 | | 1.97E-38 |
| 6 | 38681617 | | 38683616 | | DNAH8 | | 0.222 | | 7.07E-56 |
| 12 | 51421850 | | 51423849 | | SLC11A2 | | 0.228 | | 1.63E-26 |
| 5 | 140179283 | | 140181282 | | PCDHA3 | | 0.230 | | 1.62E-81 |
| 8 | 125859479 | | 125861478 | | LINC00964 | | 0.233 | | 2.51E-35 |
| 17 | 79899789 | | 79901788 | | PYCR1 | | 0.234 | | 1.15E-32 |
| 5 | 66299282 | | 66301281 | | MAST4-AS1 | | 0.235 | | 1.02E-19 |
| 5 | 1886851 | | 1888850 | | IRX4 | | 0.245 | | 1.71E-69 |
| Chromosome | Start | | End | | Symbol | | Delta Beta | | *FDR* |
| 19 | 22467710 | | 22469709 | | ZNF729 | | 0.253 | | 3.81E-68 |
| 6 | 35115888 | | 35117887 | | TCP11 | | 0.254 | | 7.73E-43 |
| 8 | 79674167 | | 79676166 | | PRKRIRP7 | | 0.256 | | 4.54E-50 |
| 7 | 153582682 | | 153584681 | | DPP6 | | 0.276 | | 1.72E-75 |
| 12 | 6307381 | | 6309380 | | CD9 | | 0.307 | | 5.22E-53 |
| 1 | 47222010 | | 47224009 | | CYP4B1 | | 0.314 | | 1.60E-48 |
