# Supplementary material for: Promoter DNA methylation analysis reveals a novel diagnostic CpG-based biomarker and RAB25 hypermethylation in clear cell renel cell carcinoma
Source: Sci Rep. 2017 Oct 27;7:14200. doi: 10.1038/s41598-017-14314-y (PMC5660223; doi:10.1038/s41598-017-14314-y)
Supplement: Supplementary file 5 — Dataset 4 [file 41598_2017_14314_MOESM5_ESM.doc]

Table S4. Methylation conditions of cg11201447, cg25247520, cg13309012 and cg08995609 in eleven cancer types

| Type | No. of Samples | cg11201447 | | cg25247520 | | cg13309012 | | cg08995609 | |
| --- | --- | --- | --- | --- | --- | --- | --- | --- | --- |
|  |  | Delta Beta | *P* | Delta Beta | *P* | Delta Beta | *P* | Delta Beta | *P* |
| BLCA | 299T+19N | -0.367 | <0.001 | -0.378 | <0.001 | -0.270 | <0.001 | -0.039 | 0.002 |
| BRCA | 719T+93N | -0.265 | <0.001 | -0.247 | <0.001 | -0.259 | <0.001 | 0.037 | 0.563 |
| COAD | 277T+11N | -0.197 | <0.001 | -0.153 | <0.001 | 0.049 | 0.275 | -0.085 | 0.001 |
| ESCA | 162T+10N | -0.509 | <0.001 | -0.448 | <0.001 | -0.261 | <0.001 | -0.037 | 0.015 |
| HNSC | 484T+14N | -0.188 | <0.001 | -0.198 | <0.001 | -0.250 | <0.001 | -0.072 | 0.012 |
| KIRP | 202T+34N | -0.266 | <0.001 | -0.229 | <0.001 | -0.373 | <0.001 | -0.388 | <0.001 |
| LIHC | 327T+46N | -0.296 | <0.001 | -0.259 | <0.001 | -0.145 | <0.001 | -0.269 | <0.001 |
| LUAD | 386T+17N | -0.432 | <0.001 | -0.377 | <0.001 | -0.135 | <0.001 | -0.210 | <0.001 |
| PRAD | 423T+39N | -0.140 | <0.001 | -0.281 | <0.001 | -0.017 | 0.176 | 0.228 | <0.001 |
| THCA | 456T+20N | -0.049 | 0.027 | -0.016 | 0.445 | -0.245 | <0.001 | -0.340 | <0.001 |
| UCEC | 390T+25N | -0.276 | <0.001 | -0.264 | <0.001 | -0.076 | 0.021 | 0.185 | <0.001 |

Abbreviations: T: Tumor; N: Normal; BLCA: Bladder Urothelial Carcinoma; BRCA: Breast invasive carcinoma; COAD: Colon adenocarcinoma; ESCA: Esophageal carcinoma; HNSC: Head and Neck squamous cell carcinoma; KIRP: Kidney renal papillary cell carcinoma; LIHC: Liver hepatocellular carcinoma; LUAD: Lung adenocarcinoma; PRAD: Prostate adenocarcinoma; THCA: Thyroid carcinoma; UCEC: Uterine Corpus Endometrial Carcinoma.
