# Supplementary material for: Promoter DNA methylation analysis reveals a novel diagnostic CpG-based biomarker and RAB25 hypermethylation in clear cell renel cell carcinoma
Source: Sci Rep. 2017 Oct 27;7:14200. doi: 10.1038/s41598-017-14314-y (PMC5660223; doi:10.1038/s41598-017-14314-y)
Supplement: Supplementary file 7 — Dataset 6 [file 41598_2017_14314_MOESM7_ESM.doc]

Table S6. Characteristics of 19 ccRCC samples from the Anhui provincial hospital

| Variables | Characteristic | No. of patients |
| --- | --- | --- |
| Sex | Male | 11 |
|  | Female | 8 |
| Age (years) | ＜60 | 14 |
|  | ≥60 | 5 |
| Fuhrman grade | G1 | 5 |
|  | G2 | 12 |
|  | G3 | 2 |
|  | G4 | 0 |
|  | GX | 0 |
| Tumor size | T1 | 17 |
|  | T2 | 2 |
|  | T3 | 0 |
|  | T4 | 0 |
| Lymph node | N0 | 18 |
|  | N1 | 1 |
| Metastasis status | M0 | 19 |
| AJCC stage | M1  I  II | 0  17  2 |

Abbreviation: No., Number.
